# Transcultural skills for early childhood professionals

**DOI:** 10.3389/fpsyt.2023.1112997

**Published:** 2023-04-21

**Authors:** Rahmeth Radjack, Muriel Bossuroy, Hawa Camara, Fatima Touhami, Anaïs Ogrizek, Juliette Rodriguez, Marion Robin, Marie Rose Moro

**Affiliations:** ^1^Maison de Solenn, Department of Adolescent Psychiatry, Cochin Hospital, AP-HP, Paris University, Paris, France; ^2^Department of Child and Adolescent Psychiatry, University of Paris, Paris, France; ^3^CESP-UVSQ, DevPsy, INSERM, Université Paris-Saclay, Villejuif, France; ^4^Unité Transversale de Psychogénèse et Psychopathologie, Sorbonne Paris Nord University, UTRPP, Villetaneuse, France; ^5^Department of Adult and Child Psychiatry, University Hospital of Martinique, Fort-de-France, France; ^6^Department of Adolescent and Young Adult Psychiatry, Institut Mutualiste Montsouris, Paris, France

**Keywords:** culture, migration, transcultural competencies, perinatal, maternity ward, pregnancy, birth

## Abstract

**Context:**

Transcultural skills are especially useful for those involved in the perinatal period, when parents and babies must adapt to one another in a setting of migration a long a focus of transcultural clinical practice.

**Objective:**

The aim of this article is to provide useful transcultural skills for any health care worker (e.g., psychologists, child psychiatrists, midwives, family doctors, pediatricians, specialized child-care attendants, and social workers) who provide care or support to families during the perinatal period. It highlights the cultural aspects requiring attention in relation to representations of pregnancy, children’s needs, obstetric complications, and postnatal problems. Taking into account the impact of culture on clinical evaluation and treatment can enable professionals to distinguish what involves cultural representations of pregnancy, babies, and sometimes of disease from what is associated with interaction disorders or maternal psychopathology.

**Methods:**

After explaining the relevance of transcultural clinical practices to provide migrant mothers with better support, we describe 9 themes useful to explore from a transcultural perspective. This choice is based on the transcultural clinical practice in our specialized department.

**Results:**

The description of these 9 themes is intended to aid in their pragmatic application and is illustrated with short clinical vignettes for specific concepts. We describe situations that are extreme but often encountered in liaison transcultural clinical practice for maternity wards: perinatal mourning with cultural coding, mediation in refusal of care, cultural misunderstandings, situations of complex trauma and of multiple contextual vulnerabilities, and difficulties associated with acculturation.

**Discussion:**

The transcultural levers described here make it possible to limit cultural misunderstandings and to promote the therapeutic alliance. It presupposes the professionals will concomitantly analyze their cultural countertransference and acquire both the knowledge and know-how needed to understand the elements of cultural, political, and social issues needed to develop clinical finesse.

**Conclusion:**

This combined theoretical-clinical article is intended to be pedagogical. It provides guidelines for conducting transcultural child psychiatry/psychological interviews in the perinatal period aimed at both assessment and therapy.

## 1. Introduction: the importance of acquiring transcultural skills

There are a thousand and one ways to think about, educate, and take care of babies across the world, and they are all equally effective in enabling good child development—both emotional and physiological. Several sociological, anthropological, and psychological works confirm this universality without uniformity (1, 2). Among the differences that may affect our clinical evaluation, we should take into account the distal interactions in the mother-baby relationship and the more proximal interactions in some cultural groups (3–5). In many cultural groups, for example, tactile and motor interactions count more than visual and verbal interactions: mothers carry babies frequently; massages shape babies, drain their impurities, and stimulate them. That is, babies are considered malleable, impure, from a world of invisible beings (6). They must be embodied and shaped for the world of humans. Elsewhere, it is eye contact and speaking that have become most important in interactions to compensate for the distal character of other interactions, e.g., swaddling to avoid skin-to-skin contact, strollers later on (1). There are numerous examples around the world of important differences in how to put babies to sleep, feed them, play with them, care for them, and address them. There is not one good way or one bad way to interact with babies.

The fundamentals, such as the theory of attachment described by Bowlby (7), must also be taken into account in the context and the environment in which infants develop, along with their mothers and the various, often multiple, attachment figures (and not only one or two people who care for the baby consistently and continuously to aid their social and emotional development).

The absence of uniformity also lies in the way of thinking of a family (8). The concept of a family is enlarged beyond the nuclear in many cultures: the opinion of a maternal uncle in a matrilinear society (such as Haiti, some regions of Indonesia, and Cameroon) about a child’s education can be more important than that of the father, for example. In other places, the role of the elders and even the ancestors is important (Asia), and children belong more to the group than to their two parents.

These differences create the need for professionals to decenter, that is, to not interpret the unknown in terms of the known, for that risks eroding human complexity and inducing cultural misunderstandings. On the contrary, we must leave room for doubt when we are faced with ways of doing things that seem surprising to us.

Knowing how to adapt the framework and the questions in an interview with patients or clients allows us to gain access to their different cultural representations. In our health care departments in France, the republican ideology is supposed to protect against the risk of pointing out differences in patients from other cultures and other religions, so that patients can feel they are receiving care with equal rights and without either judgment or exclusion. These universalist positions avoid any insistence on the difference between communities or cultural groups (9). Others nonetheless underline the need to recognize the specific needs of minorities and to find appropriate responses to these needs (10–13).

The curriculum allocates very little space to the consideration of cultural elements in health care relationships (14). Care providers are often very prudent, even inhibited, about approaching cultural questions, sometimes to the point of being implicitly reluctant to use an interpreter in situations where a language barrier exists, although this can be considered to constitute a form of institutional abuse/mistreatment (11). Another pitfall has been observed in France: the logic of integration that is supposed to erase or at least erode these differences led professionals of early childhood to advise in the 1980s that the use of French should be privileged in personal family spheres. One consequence of this was an impairment of the language quality in family interactions in non-French speaking families. It is clearly recognized today, however, that using a mother tongue other than that of the host country does not slow down the acquisition of the host-country language. On the contrary, the more languages one learns, the easier it is to learn others (15).

The objectives of this article are simultaneously theoretical, clinical, and pedagogic to demonstrate the importance of taking these elements into account to obtain a better clinical understanding and evaluation of the situation in the perinatal period and thus be able to provide optimal support. The aim is to help care providers to acquire/adopt transcultural skills, that is, an overall set of aptitudes and attitudes that allow them access to these essential elements of understanding. Academic training is needed to impart transcultural skills to early childhood professionals, regardless of their discipline (16–18). Transcultural skills consist in adopting a dynamic posture toward the acquisition of skills (such as knowing how to work with an interpreter, to adapt one’s manner in conducting an interview, general culture) and attitudes (nonjudgmental, and nondiscriminatory toward minority populations, decentering), which help to take the impact of culture on symptoms into account (19). The clinical evaluation is thus more detailed, without creating the feeling of stepping past the lines of our republican institutions. We know that these cultural elements suffuse/pervade/dominate/invade the thoughts of hospital patients, in particular during this period of pregnancy and childbirth. Tact is needed to show the usefulness of this exploration and its benefits for clinical evaluation, treatment, and support of mothers in the care related to mothering.

## 2. Implications for the clinical situation/for clinical progress: value of the transcultural approach in the perinatal period

### 2.1. Psychological vulnerability

The concept of psychological universality and of universality in ways of mothering a young child makes it impossible to label the way something is done in one culture to be the best way of doing it in any culture or to define any vulnerability as greater in the babies of a given cultural group. On the other hand, outside of their cultural group, families can find it distressing to give birth in a foreign country. Migration can induce a loss of bearings and undermine a sense of parental competence. Confronting different mothering techniques and questioning themselves about how to raise a baby born in a foreign land are factors that make parents especially vulnerable during this period. Babies themselves can appear strange and foreign to their parents’ eyes simply because they were born in another country (11). What will their parents be able to transmit to them of their cultural values, of their identity? Migration, especially if recent and if the couple or the mother is isolated, induces a sudden break from previous reference points (3, 4, 20, 21). The absence of a word, a practice, of support at the start of their baby’s life/at the start of their life as parents—a moment when the group is primordial—can make isolated parents still more insecure (20). That is, numerous cultural practices exist to protect mothers and babies and to enculturate babies: for example, among Hindus, both naming according to specific astrological criteria and a head-shaving ritual for purification. In several non-Western cultures, the mother must comply with a period of rest and social withdrawal after giving birth. During this time, the baby is presented to the world and the mother is released from her domestic tasks, surrounded and supported by older women. Pregnancy and childbirth are among the life stages that require rites. Among these, Van Gennep (22) distinguished rites of protection from rites of passage. The first are intended to protect the mother and child from perinatal risks but also from dangers and the malevolence of both the visible and invisible worlds. The rites of passage, on the other hand, make it possible to “forbid, from birth, even pregnancy, the fusion of the mother and the baby, to unify the body and the mind or soul (the psyche), and to manage the anxiety of death inherent in humanity” (23). The childbirth is thus marked by a period of reclusion (margin period), followed by rites of reintegration and a social return from childbirth, which is the moment of both the mother’s churching ceremony and the baby’s second (psychic) birth (23–25).

This planned isolation period of 40 days exists in several cultures, not solely in Muslim areas. In the words of Ben Mohammed (26, p. 184) about North Africa, “the 40 days following the birth of a child are particularly dangerous for the mother and child. The tomb remains open during that time.” The mother–child dyad is isolated, especially during breastfeeding, so as not to attract “the evil eye of envious women who might make the milk go dry.” Other mothers have stated that in China too, the rule is “confinement” for a month after giving birth: neither the mother nor the baby is allowed to leave the house, except for medical appointments. And in this case, the mother must cover her head. She must also avoid tiring activities such as reading or watching television and ideally, she must stay mostly in bed. She must avoid washing her hair and therefore must settle for a quick sponge-down with hot water and lemongrass oil. At the end of the month, a big party is organized, bringing together all family members.

Distance from the country and the absence of the group can make these practices more difficult. Some families may feel insecure about whether they have performed these practices correctly. Whether or not we are superstitious, we are carried by a collective unconscious, and we in the West also have our own beliefs without always realizing that they are cultural and not universal. We need to feel that we belong to a group at moments of vulnerability or during life passages. This cultural dimension can become important in particular when there is a problem or an illness. Inversely, the fact of feeling active is protective and can help a parent to emerge from a traumatic shock. It is not unusual to see a mother or an isolated couple frozen due to a recurrent trauma or just by the fact of not knowing, not recognizing, or not daring to know how to express it because of the language barrier and the cultural distance from the professionals attempting to offer support.

Sometimes, in the absence of the group, only the father is present; he may find himself in a place that is not supposed to be his. In some cultures (25) it is even bad luck for the father to be present at the birth. When he finds himself invested and involved in mothering the newborn, the risk is that he may deepen the gap with traditional mothering and induce guilt and a feeling of incompetence in the mother (27). In these situations where the initial milieu is traditional, what is missing is other women (cousins, aunts, and grandmothers), co-mothers and especially the mother’s mother. Rodriguez describes this particularly for cesarean births to migrant women (27). Some studies nonetheless qualify the ambivalence of this place: the presence of the father alone may mitigate the mother’s feeling of isolation and reduce some of the risks associated with it. Lameche (28) described this in a study of a newly arrived North African population. The report RéMI related to the cesarean rate, which appears to rise less when the partner is present (29).

Forms of breastfeeding can also be transformed during migration: faster passage to mixed breastfeeding, because of preoccupation about the need to work and make a living as rapidly as possible. Some mothers say they fear the child “will get used to being carried” and avoid doing that frequently because there is no one else to do it (30).

### 2.2. Vulnerability that can be transformed into psychopathology

The risk of psychopathology is greater among migrant patients, especially for pre- and postpartum depression, which affects, respectively, 38 and 50% of migrant women, with a higher risk for more recent migration (31–33). Complex post-traumatic stress states are also overrepresented (34, 35). In the first place, we often find in clinical pictures of complex PTSD impaired trust in others. That is, trust has been endangered in repeated, intentional episodes of trauma. Dissociative symptoms, somatization, and feelings of hatred and anger are also frequent in the general migrant population. The repetition and/or reactivation of traumatic events contributes to them. The traumatic valence of a migrant’s journey may be found at different three time periods: before migration, during the voyage, and after arrival. Before the migration, some departures are associated with traumatic situations, civil war or conflicts, or a flight from complex social or family conditions. Some mourning or trauma can recur, sometimes months or even years later. The period of pregnancy is propitious to the emotional reactivation of these unresolved past events (3).

During migration, the journey can be affected by trauma. Some migrant women traveling alone experience sexual violence during their travels (36).

Afterward, the living conditions in the host country, sometimes precarization, the waiting time to the regularization of their socio-administrative situation, job hunting, and looking for housing can generate discouragement, anxiety, and disillusionment about the initial migration plan. Some symptoms are directly linked to precarization or isolation from relationships (37, 38). Micro-aggressions can also have an impact^1^ (39). Finally, very preterm birth or obstetric complications can present the risk of the infant’s death or disability and have a traumatic impact on families.

### 2.3. Physical vulnerability

In France, the number of at-risk pregnancies, cesarean births, and preterm births appears higher among migrants, particularly those from sub-Saharan Africa (31, 40, 41). Nonetheless, the literature on this point is not entirely consensual (42). The factors advanced to explain the excess risk of cesareans among migrant women are limited and inadequate. It could be linked to insufficient access to prenatal care, representations of pregnancy follow-up that are different from those of the country of origin, communication barriers, and often precarious and inhospitable conditions on arrival in the host country/France, fragile maternal health, a high body mass index, feto-pelvic disproportion, or even simply isolation (40, 43–46).

The precarity associated with migration is an important perinatal risk factor, as are isolation, absence of marital support, administrative status, and social insurance coverage (31, 45, 46).

## 3. Actionable recommendations: the interview in the perinatal period

Our transcultural team is composed of psychologists, nurses, and psychiatrists trained in transcultural clinical practices with Professor Marie Rose Moro, a leader of transcultural psychiatry in France, specialized in the clinical care of métissages (cultural mixing) in babies, children, adolescents, and their families.

We have deduced interview themes that allow a transcultural approach in child psychiatry during the perinatal period. These levers come from our diversified clinical practice applying transcultural methods, adapted and personalized to the situations for which this expertise is required. These include liaison work in the hospital, discussion and support groups with pregnant women or recent parturients (47, 48), transcultural consultations with a group of co-therapists from various cultures (3, 4, 49), mediation at the hospital with an interpreter-mediator, and institutional work (team supervision, university instruction or professional training programs, and reflexive interventions in multidisciplinary staff meetings). These guidelines also consider the bases of ethnopsychoanalytic theory (50), combined with approaches described in the international scientific literature (4, 14, 16–18).

### 3.1. Start the interview by creating the basis for an appropriate relationship

The interview’s framework presupposes establishing the conditions for a balanced and horizontal relationship. The interviewer exits the position of expert and instead shows a posture of cultural humility (51). Sitting in a circle, sometimes sharing some water, can facilitate this first encounter (52, 53). Showing an authentic interest in the other’s culture and representations can transform the relationship. This is done by asking in detail about their cultural identity, beyond their country or nationality. What are the family’s cultural affiliations? What language do they speak? What city or village do they come from? Language is an important aspect of identity. Exploring the languages the patient speaks is often the occasion to praise those patient who are multilingual for this accomplishment. Both ethics and quality of care require finding an interpreter to ensure mutual understanding; it is thus necessary to identify the language the interpreter should speak. The help of family or friends has its limitations for announcing bad news (for example, a suspected malformation on ultrasound or complications in neonatology). Thus, partial mastery of a language cannot suffice in these contexts to ensure mutual understanding, especially as there can be an initial level of stunned speechlessness even in their native language, it is often necessary to repeat news that is difficult for everyone to understand. The interpreter, who is often someone with whom the patient can identify, enables the mother to assert herself, to not fear being judged: the cultural distance is thus reduced, and the interpreter facilitates bridges between the two countries by knowing the codes to both worlds. Finally, the expression of emotions is often facilitated by use of the mother tongue which, in turn, facilitates the narration [Ricoeur’s narrative identity (54)].

Sometimes reluctance linked to financial cost or time are mentioned, or those linked to individual or collective unconscious factors. Studies show that the use of interpreters and mediators offers cost benefits for public health (55). It is important for therapists to show that they are making an effort to adapt to and respect the patient by seeking to share the same language.

The notion of hospitality in the welcome is also important, for it can contrast with the exterior world, sometimes associated with the austerity of migration policy (56). The tone of voice is almost as important as the content (57).

One of the principles of receiving patients in a place providing care is to create a relationship of trust, which is all the more important for women who have experienced intentional and repeated traumas. For that reason, it is important to explicitly state the meaning and nature of a “talking” therapies, of seeing a psychologist or psychiatrist, without this being associated with the mental illness of the “asylum” sector. Finally, the role of each professional must be clearly explained, especially for more highly medicalized pregnancies. The psychological support must be related to the risk of vulnerability, while assuring the patient that there is no association with a severe overt mental disorder. The families may not be able to understand the notion of interaction disorders unless the therapist describes them clearly.

Finally, one way to show that it is possible to approach the cultural dimension in the framework of a medical interview is to begin the interview by self-disclosure, that is, short personal revelations (while remaining professional) used to create empathetic understanding: they may concern hindsight about the experience of other patients or be a personal shareable and generalizable experience.

This self-disclosure aims to create cultural proximity (if the interviewer is comfortable with it), but also to demonstrate the diversity that authorizes patients to assume their culture and thus to be able to refer to it as a resource, to subjectively structure an issue. For example, near the end of an interview during the perinatal period: “Where I’m from, in North Africa, we organize this protection, that birth ritual, when a baby’s born; and where you come from?” This type of interview sometimes humanizes the professional and promotes the therapeutic alliance.

### 3.2. Elaborate a narrative that mitigates the risk of migration cleavage and considers the possible traumatic dimension (re-establish continuity between before and after)

Child psychiatry and transcultural psychiatric interviews are often based on narrations (3, 5, 11, 52). The therapist accepts and welcomes the cultural narratives that serve as a container for the intimate individual material relating to the current situation of the mother, family, and baby.

Bridges are built between life “back there” and life here to avoid the risk of a migratory divide and to share cultural representations. Indeed, parents often tell their story starting from the day they arrived in France. If we do not actively look for information about their premigration history, it is impossible to have access to it. This reaction can be linked to the reading of migration trauma, with too great a separation between before and after. The cleavage of migration is a phenomenon described by Nathan (58): the experience that every migrant has of mental separation between before and after migration. It resembles the loss of a psychological and sensory envelope with which the migrant decodes the exterior world and stimulates the need to create another one, like a new skin.

Evoking the patient’s migration experience, its determinants, its course, what she thinks of life before and after migration—these questions make it possible to help her establish a continuous narrative of the different periods of her life. Often the heroic valence of the journey is manifest in this account and deserves to be underlined for therapeutic purposes. These elements must be explored progressively; they can finally allow the patient to recover a feeling of the continuity of existence, the continuity of her existence (the *self-continuity* described by Chandler and Lalonde (59)). We search constantly for coherence in their pathways, so often characterized by family separation, cultural ruptures, and changes in their care. This coherence and this life story are necessary to cross/bridge the passages of life (52). In the face of this disillusionment and sometimes precarization, it is useful to ask about her professional identity, her education, her place in the family before migrating, and to recall who she is. Sometimes disillusionment and/or trauma seem to deprive the journey of its meaning. This must be reactivated to give her the strength to continue. Contrary to what is often thought, asking about the meaning of the journey or exploring the other’s cultural affiliations is not uncomfortable when the therapist’s interest is authentic. Finally, it is necessary to welcome disillusionment, to share the disarray sometimes, often to help actively on the social level, assuming this professional role, despite being a health care provider. That is, a patient cannot engage in psychotherapeutic work until her material survival and shelter are assured. In pregnancy or at childbirth, this need for a safe place can become obsessive and constitute a form of primary material preoccupation together with a primary maternal preoccupation (constructing a home).

She must be restored to her story both gradually (60) and prudently to take her own rate of progress into account: the traumatic valence of a journey exposes her to the risk of reactivation and emotional overflow. Time is needed to transform and develop these aspects at a psychotherapeutic level. Putting it into words—into a narrative/story—is sometimes painful. Regardless of the country one leaves, regardless of the reasons for leaving, there is inevitably a loss causing narcissistic injuries the extent of which is sometimes difficult to measure, but also, simultaneously, producing unsuspected gains (13).

### 3.3. Reconstruct the networks of belonging

On a practical level, the networks of kinship and social belonging are reconstituted during the interview: Which family members stayed behind in the home country? Who in the family is in France? What contacts do they have (telephone, frequency)? Has the baby’s birth been announced? Are there any friends, people from the community, at hand? Is the parent able to speak his/her mother tongue? Even for the most isolated individuals, encouraging them to talk about family during a consultation, helping them to telephone family members, or encouraging them to do so, can help them feel less lonely, and loneliness is a very deleterious feeling during the perinatal period.

Do not be afraid of mentioning the people missing in France; on the contrary, make them exist in the session: the grandmothers, the child’s father, even the children who remained behind. Prevost et al. (61) shows for example in her qualitative study of the experience of migrant mothers giving birth in France after leaving a first child behind in the country she left that there is a massive reactivation of the missing child during the new pregnancy. Often the women have left them to protect them and to bring them over later, once the material conditions are correct. Professionals may often not dare to raise the question of the children left behind for fear of making the mother feel guilty with anxieties about having abandoned them. Some professionals are also comforted by the idea that culturally favored entrusting of children does not engender distress. Recent studies point out the consequences and intrafamily relationship stakes that it raises. Even though this practice is recognized and valued in society, it is not without risk of psychopathology for both mothers and children (61, 62).

Finally, taking the cultural place of the extended family into account sometimes makes it possible to adapt the hospital’s framework. Knipiler (63) described how the experience of a neonatology unit stay aggravates the experience of isolation and how the extended family can be allowed in neonatology departments that are aware of the limits of the concept of the nuclear family in Western areas.

The interpreter can also function as someone with whom the patient can identify; this can also help her to escape her sense of isolation. Finally, therapists/care providers/professionals can creatively employ local groups and associations. They can also discuss with the patient the difficulties of contacting the family, despite today’s social networks which make it easier than in the past: these difficulties sometimes follow traumas (and/or the desire to protect the family “back there”). Finally, when mother has had not news of her family or friends because she had to flee rapidly, referring her to the Tracing services provided by the International Red Cross^2^ can spark new hope.

### 3.4. Help her to escape a feeling of powerlessness and facilitate her psychological reinvigoration

Therapists faced with the patient’s psychic exhaustion from confronting recurrent adversity must often take an active position, adapted to the patient’s tempo of progression to escape their own feelings of powerlessness. We help to coconstruct solutions based on what emerges from the narrative, facilitated by the interview levers described here.

- An example of a woman’s prenatal mourning with an acute traumatic reaction

*Joëlle arrived in France recently. She came from the DR Congo and was hospitalized after her delivery. One of her twins died in utero. The other twin is alive, but Joëlle is stunned and does not seem able to succeed in paying attention to him.*

*Joëlle explained that she cannot either eat herself or breastfeed the baby because the other twin is "out in the cold and frozen" an allusion to the morgue/mortuary. Her principal preoccupation was that "he's suspended between the world of visible and invisible beings," the latter being, in other words, the world of the dead and spirit creatures. She understood that we were not judging her and that we would not hesitate to talk about different cultural representations of the pregnancy and ontological representations of children. She understood this because of our explicit questions about how to welcome a child into the world, what birth rituals should be done, what mourning rituals were needed. She could not be primarily preoccupied with the living baby as long as his twin was not buried in due and good form. Moreover because the two were linked, neither could be appeased and recognized in the world of the living or the dead. But Joëlle's very great precarity, her isolation from family and any community (because she had arrived very recently in France), and her lack of knowledge of the organization of funerals in France aggravated her experience of isolation and prevented her from acting.*

*Helping her to escape this sense of isolation was the first step to re-energizing her. First, we talked about her family. Even thought they were back in her country, far away, we made them re-exist in this interview. She said she had not been able to tell her mother, to whom she is nonetheless close, of her delivery, both because she lacked the money to make the call, but also to protect her. Wouldn't her mother herself feel helpless, even sad about Joëlle even as she was in her own difficult situation in RD Congo, a situation that Joëlle had fled precisely to be able to later help her family? In every culture, mourning is shared by the community/collectively. Culture surrounds/lines these difficult life passages to generate meaning and master the violence of the unexpected. What can be done when the person is far from her cultural group? We thus lent Joëlle our work telephone so that she could call her mother and connect with her and her country. This simple act probably prevented the prolongation of her hospitalization. The next day, she told us that her mother was planning to organize a ceremony there, with the help of the extended family, the day of the funeral. We obtained information about - and told her - the specific date this month of the burials of fetuses and newborns who died at the maternity hospital, and where it would be. There is a support system covering the costs of burial for families in situations of great precarity and/or who do not want the burden (fetuses who died in utero or medical terminations of pregnancy). We also informed Joëlle that she could see a priest at the hospital.*

Sometimes, it is other mothers who help the patient to find solutions and to escape from this psychic paralysis, even in the most extreme situations (very great precarity, extreme preterm birth). We have observed this in particular in support groups around cultural questions about welcoming a child (30, 47, 49). These groups help to mitigate the feeling of isolation and constitute a creative and relevant form of care at the maternity ward. We can cite here a conversation around African massages. The practice was relegitimized in one support group (47). One mother thought the practice was forbidden here, that the vigor involved could be badly interpreted by professionals. This led another to say that she would have liked to do this, that it was important to her, but she really did not know how to do it well, because it is grandmothers who handle the initial care after birth “back home,” mothers being considered too gentle/subdued to do these massages. Hearing this, another mother from a related cultural group improvised the massage gestures on her baby for the rest of the group.

### 3.5. Promote harmonious cultural métissage

One of the coping strategies described in the literature (64) is the maintenance of continuity despite an unstable and shifting situation. It is thus desirable to facilitate access to familiar things—things the patient knows—to help her regain her bearings. This enables a transition to integrate the values of the host country more easily and to construct a harmonious cultural métissage or mix between two cultural worlds. The progressive learning of new cultural norms, as well as the maintenance of religious beliefs and practices within the host country are part of the strategies for achieving this construction. Links with peers from the cultural community already more accustomed to the host country and who have often endured identical or similar situations can play a major role, as sharing experiences mitigates isolation and discouragement. A double support network, in the host country and the country of origin, may have a protective effect on the mental health of migrants (14, 64).

A harmonious cultural métissage protects against the stress of adaptation to the culture of the host country. Metissage is an identity building continuum (or a set of processes) that integrates the feelings of belonging to many cultural universes. It is found among children from mixed couples or couples from migrant backgrounds or among child migrants, who tend to adopt progressively some of the cultural norms of the adopted country.

These involve maintaining a feeling of belonging to the original culture, while adopting new affiliations in the host country—all as part of a creative process integrating the values of each culture. Acculturated individuals^3^ may thus be the most highly exposed to this risk of psychic vulnerability (65). That is, we encounter in our everyday transcultural clinical practices couples or isolated mothers whose sense of identity is sometimes lost due to their cultural mixing.

Behind their resistance or their lack of knowledge of cultural theories, we often see a willingness to understand the cultural sense of the symptoms of their baby or child, leading them to rethink the disease from a cultural perspective, because medicine has not led to recovery, or because the coding of the symptoms clearly evokes a cultural interpretation. Often this occurs only because of the patient’s cultural group, and one of the means of treatment is precisely to link it to the culture of the country they come from, in one way or another (66).

Within a single country, the effects of globalization and accessible media and social networks engender rapid transformations. Knowledge is mixed in a single generation, which is evidently valuable, but is also a loss of bearings and identity across a single generation. The painful moments of life (a chronic disease that medicine cannot cure) or its passages (birth, death, becoming an adolescent or reaching adulthood), when they are difficult, sometimes call for cultural readings.

### 3.6. Construct a narrative of filiation

It is also our goal to attempt to elaborate a narrative surrounding the child and to develop a form of narrative of parenthood (67), thereby placing the child or the future child within the family history. The focus is also on the notion of a cultural mix or blend: help with the construction of self between the two cultural worlds the patient belongs to and the creative use of the wealth from both (without this appearing incompatible). “There is no filiation without putting a history into words, without a narrative, without a narrative on filiation” (Golse & Moro, op. cit.). Putting the baby’s history into words is paramount. The feeling of containment, protection, and roots procured by the family and the entire group of belonging is primordial because it enables the mother and child to be part of an intergenerational web—filiation and affiliation (68–70).

In the perinatal period, asking about the history of the child’s name makes it possible to identify first of all who he is and often leads to an important story of his history and his parents’ investments. Given the concept of an “exposed child” at risk of vulnerability because he is the first to be born in France, this work of making him a history is very important. It also reduces the risk of reciprocal feelings of strangeness.

Without the group, it is sometimes difficult for mothers to achieve this, because of the massive nature of their worries, their numerous disruptions, exposing them to a risk of “mental blank,” withdrawal, silence, and encryptment. Therapists can sometimes find themselves in the midst of extremely problematic issues: the reaffiliation of a baby born from a rape, which can be thought of/handled in several ways: by naming, or through religion, membership in a matrilinear line or a new mixed cultural group, etc. (71). In these cases, we sometimes use a valuable narrative support, the transcultural genogram (72).

It makes it possible to represent families who come from elsewhere, taking into account migrations as well as blended definitions of the family object in its psychoanalytic and anthropological sense. It thus provides support for the narration of family stories for migrant families who sometimes find it hard to conceive and transmit these bonds.

### 3.7. Make the family the expert, decenter oneself

At all of these stages, it is essential to adopt a decentered posture (distancing oneself to avoid ethnocentrism) in particular when one has a doubt, a feeling of strangeness, of missing the content of the clinical encounter. To learn from the family, it is important not to fear facing otherness. Assuming that some things are not spoken of/kept quiet, we recommend showing (and thinking) that you are ready to listen without judgment.

The interview is based on co-construction: the patient is given an expert role and a mother is invited to express herself the way she wants, to be fully heard, avoiding turning what is unknown into what is known. This can be done through narratives such as: “what is usually done in your country to welcome a baby? What are the rituals at birth?” The mother can therefore become an ambassador for the collective or family knowledge that she wishes for and chooses. What is most important is the meaning for her of whatever cultural or religious practice she decides to honor, as migration is bound to trigger changes, choices, and mixing. It is important to avoid the pitfall of cultural generalities: for example, that a woman who comes from a rural area does not say the same things as a woman from the city.

Finally, we aim to co-construct solutions: “even if we cannot do exactly the same as in the home country, something can be done, for instance, asking the family to carry out part of the ritual over there,” or else over here, even if it is not on the day initially required, or carry it out symbolically and partially. Culture is a dynamic process, constantly changing, and certain adaptations in practices on foreign land are perfectly valid.

This approach often helps people to escape a feeling of powerlessness and enables skills to emerge despite a traumatic past.

Here we cite the example of African massages of babies, or the technique of putting Tamil babies to sleep with rapid, jerky movements, the tonicity of which can surprise the untrained eye (1). The error of interpretation would be to consider them to be shaken babies, even as the techniques of this form of mothering, transmitted from generation to generation, protect the baby from any shock. The transcultural posture consists, on the contrary, of leaving room for a tolerable doubt: leave yourself the time and give yourself the means to apply the procedure used in complementarism that combines a (non-simultaneous) anthropological and psychological/medical reading (73). Therapists must be capable of listening non-judgmentally and non-ethnocentrically, even if they do not understand.

- A Kurdish family and its very preterm baby

*Elif, a young woman of Kurdish origin who arrived in France shortly before giving birth to a very premature baby. Her social and economic situation was precarious. She did not speak French. The professionals in the neonatology department were astonished because the she was discharged but has not been to visit the baby for the entire first week in the department. The parents did not understand the hospital staff's explanations. They could not be reached by telephone. The team was feeling alarmed and powerless about the situation. At the end of a week, the parents returned with clothing and a stuffed animal for the newborn. They were accompanied by a friend who interpreted for them and the staff. They then explained the status of babies in their culture. For them, babies do not yet belong in the world of humans when they are born. They are suspended between life and death, between the world of the visibie and the world of the invisible. The parents were therefore not yet authorized to meet them. Now, after seven days of life, he can be named and welcomed to the world of humans.*

It is not rare for several parents from diverse cultural minorities to say that they are not coming to see their very preterm child because he does not belong to them yet. They entrust the infant confidently to the medical world. This does not at all prejudice their future investments in the child.

### 3.8. Elaborate a meaning

This transcultural posture allows access to cultural representations and sometimes to etiological theories. These cultural theories (74) give meaning to distress or suffering or a difficult event and may offer comfort beyond the medical cause or even allow the family to take action and thus contribute to the child’s wellbeing. It is frequent to see families give a cultural meaning to the chronic disease of a child whom medicine cannot cure or to explain a death, a singularity. From this sense of/search for meaning comes a cultural therapeutic logic that allows patients to overcome their feeling of powerlessness. They can for example, rename a child, provide a dowry for the marriage of unmarried parents, recognize the child’s ontological status, and perform the appropriate rituals to keep the infant in the world of humans. For example, the ontological representation of autism in West Africa can enable parents to consider this singularity acceptable (and culturally coherent). In some cultural groups, when a person is unwell, an external cause is sought (attack by witchcraft, badly named child, unprotected family alliances, unpaid dowry, etc.). To harm a group or a family, the most vulnerable in the group can be targets, a child, for example. Culture intervenes to enable thinking and healing, by allowing action and mastery of the associated anguish by rites of holding, cultural protections, and by giving meaning to the meaningless (75).

In some situations of unsuccessful treatment, this development of these etiological levels and anthropological treatments can take place in transcultural consultations in a department offering therapist groups with a flexible number of therapists trained in transcultural clinical practices (3).

Some interview questions can help to develop a narrative around the elaboration of a meaning: how is a child welcomed in the home country? How would the problem of a pathological pregnancy or a maternal psychological pathology be described? What would be done? How would the problem or disease be named? Have others been harmed and what could be done (or wasn’t done that is possible today)?

There again, the etiological theories must be considered in a context and cannot be generalized: they depend on the cultural group (or groups) involved, the relation to migration, what the combined symptoms suggest, the family’s singular history, and the individual and collective knowledge of these situations. They may be part of a global framework of intergenerational distress, without an association with a theory for a specific disease.

- Refusal of a cesarean for a “sleeping baby”

*We were called into the delivery room for a young woman from Somalia, Faduma, to attempt to resolve a situation in which she was refusing a cesarean, delivery despite the risk of fetal death, with an argument based on cultural elements. Faduma's partner, the father of the unborn child and her only source of support in France is also opposed to a cesarean.*

*The medical team informs us that the interviews have thus far been conducted through a staff member who speaks Arab, as Faduma does not speak French. Faduma has been in France for only months; she fled her country for reasons related to family trauma (fear of a forced marriage, with vague notions of excision mentioned). In her journey, Faduma crossed Libya and learned Arab. She was also violently raped. Because she was post-term, labor had been induced several hours earlier. The fetus responded poorly to the induction and appeared to present fetal distress (heart rate decelerations). The cervix remained too closed to allow vaginal delivery.*

*On arrival, we decided to sit down at her level. We established a framework at a pace contrasting with the context of a life-threatening emergency. We used mediation techniques, including an interpreter in Oromo, a language which the mother spoke more fluently than Arab; given the emergency situation, this professional interpreter worked by telephone. In the presence of the midwife and the obstetrician, we retranslated the medical reasons and the risks and also translated for them her precise cultural logic (see reference mediation).*

*What we learned during the interview in Oromo illuminated three essential points:*

*On one hand, Faduma insisted that she could not be mistaken about the date of the child's conception. Was she insisting that her partner's paternity was unquestionable paternity and that she had no doubt that she did not conceive during her rape in Libya? Might it not influence the fetus's fate if the conception resulted instead from rape? She thinks she is at term especially as she feels a discordance between what the monitoring is saying and her own physical sensations that she wants to interpret. She says she is attached to traditional techniques of childbirth. According to her religion and practices, prenatal care should not be too medicalized (and she was identified only late at the maternity ward) and one does what "God has decided." Through ignorance, she said, she accepted an epidural, and it was preventing her from perceiving the painful contractions. She felt that the baby could still wait to come out "naturally" and thus refused the "surgery." When we asked her to describe what she thought the surgery is, a second point of understanding emerged: she considered it similar to a "long laceration," with a statement that reactivated the experience of her infibulation*^4^ *but also the fear of disinfibulation associated with a cesarean.*

*Finally, she explained to us an ontologic conception that can make sense of a pregnancy that can sometimes last a long time, well beyond 9 months and can nonetheless be considered at term. This ontologic theory of the "sleeping baby" is culturally coherent and is known in several cultural areas, including in North Africa and sub-Saharan Africa. It developed to make the inconceivable conceivable at a time when infant mortality in those regions was high: the same fetus who left for the world of the dead during a miscarriage or an in utero death can return several months or years later.*

*This transcultural mediation took place in a particularly benevolent ambiance: Faduma only spoke when we assured her that we would not force her to have the cesarean and that we would respect her decision. The obstetrician took her traditional representations seriously and responded clearly and appropriately to Faduma's precise fears. She thus reassured her about the type of surgical incision. She also answered Faduma's questions, including her worry that the fetus's poor state was because she could not eat (concern related to the infusion).*

*Can we represent the experience of strangeness/ foreignness? Of cultural distance? Seeing so many "scopes"… when the pregnancy can be hidden to protect it (from the evil eye).*

*She and her partner finally agreed to the cesarean, and their first interactions with the baby went well. The physical health of both mother and baby was also good.*

Other mothers mentioned that cesarean birth is viewed as a cultural, religious, or even sexual transgression. They risk being repudiated by their husbands, branded as sterile, and accused of the baby’s malformations (76).

We note that anthropological studies about the perinatal period and obstetrics in traditional rural settings report negative cultural representations of cesarean births. Cesarean birth is most often feared or refused because it demonstrates to the community the mother’s inability to give birth/to know how to give birth (77). A woman from a city or somewhere else would not necessarily say the same thing.

### 3.9. Show that she has mothering skills and be careful not to quash them

A favorable outcome in the interactions between parents and babies is most likely when the parents’ skills are underlined rather than criticized (3). This is especially important when migration has damaged these feelings of competence, for the reasons cited in part l. The logic of mixing ways of doing something or welcoming other ways of doing it requires the professional to have the tact to not impair these feelings. This requires pointing out where the mother can genuinely feel competent, but also recognizing the difficulties that others would also legitimately have, by contextualizing the situation.

- Avoiding cultural misunderstandings with a traditional Bambara woman from Mali

*Aminata is a young Bambara mother from Mali, referred for a transcultural consultation by the staff from the maternal center*^5^ *that initially sheltered her and her baby. At the time of our evaluation, the baby, Ramata, was a year old and she had been placed in a foster family. Some developmental delay was beginning to be noticeable. Ramata was growing up a different language environment than that of her mother, who had arrived in France soon before her birth and does not speak French. Aminata reported that her daughter was eating better when she was not present, partaking of little jars of baby food, whose composition was unknown to Aminata.*

*Aminata described in a consultation her experience of the birth of this very preterm baby. The meeting with this baby arrived too early and was difficult; she was a stranger to her. Aminata had no representation of preterm birth/did not understand what preterm birth was and seemed stunned by the situation. Added to that were her language barrier and a life path that seemed studded with multiple traumas. Under these circumstances and the various misunderstandings between Aminata and the different teams caring for her, several professionals suspected she had cognitive difficulties. This led to a request for a transcultural consultation.*

*The often chafing cultural distance was a source of misunderstandings on several occasions, in particular on the subject of protecting the child. When meetings with Aminata resumed in a larger space and in Bambara, her native language, this young mother's explanations made sense and seemed legitimate. For example, her representations of foster care enabled Aminata to accept assistance from the child protection services easily without understanding that she would not be able to control how frequently she saw the baby. Ramata is growing up in a cultural milieu different from her mother's, a fact that deepens the latter's feeling that her child is strange/foreign and incomprehensible. To feed her child well, as s/he did not gain (but did not lose) weight at least during the winter illnesses, she fed her/him as much as possible, but did not succeed in following the hospital's dietary prescription. She was also very anxious when the baby physically vulnerable because of his preterm birth, had a fever, because in her country, people see doctors for a fever, which they associate with a serious disease. She relied on the help of professionals during this period, but without any feedback from them. They were unaware of how worried Aminata was, and of how she felt progressively dispossessed of her maternal role.*

*The staff at the center interpreted the difficulty in their meetings to be due to a poor affective investment in the maternal relationship and her difficulty in bonding with Ramata. But did this not result from a separation of mother and baby that occurred too early? The staff's difficulty in decentering and their worries led the team to read her behavior through a prism that impaired her feeling of maternal competence. The misunderstandings concerned how she carried the baby, which they described as too "tonic", her failure to attach the baby to the stroller, his sleeping in her bed … Also some of the naive unfiltered questions she asked, due to her level of trust in these professionals: such as her mention of excision which she was not planning to do because it is forbidden in France, but an issue she wanted to raise related to including her child as part of her extended family, as her aunt was a blacksmith and exciser… In this situation, we shared an exercise in decentering with the team. Everything described here was normal in Aminata's culture. There are no cradles in African villages Babies are carried proximally. Is it Aminata who is wrong, or is it that we do not understand the complexity to which we are exposing her with our tools, such as cradles? How can we ask her to decipher prescriptions that would be complex for us as well when she cannot read? How can we make her understand the concept of contextual danger? If we say, "no, not like that, it's dangerous," the harsh word erases all of her maternal skills.*

*Moreover, what is dangerous here is not necessarily dangerous elsewhere in another context.*

*We need to ask ourselves how we can explain what seems to be inappropriate without erasing her maternal competence. In exploring in detail Aminata's pathway through her narrative facilitated by the transcultural levers described above, we understand that Aminata comes from the countryside, that she is isolated and very far from her points of reference, but also from our points of reference as professionals.*

## 4. Discussion: how can cultural misunderstandings be resolved?

All of these postures and interview themes must allow an evaluation of the situation and a detailed understanding, while simultaneously avoiding misunderstandings and starting therapeutic work from the outset by a narration/story. Every stage requires a therapist’s demeanor and knowledge of clinical practice and of the complex journey of migrants.

### 4.1. Professionals must take their cultural and trauma-related countertransference into account

Cultural countertransference (78) is the set of emotions, thoughts, and attitudes felt by the care provider toward the patient, related to the cultural similarities and differences of each. Professionals are influenced by their own identity, history, and knowledge, but also their prejudices and the conventional wisdom that constitutes implicit bias (79): their religion, education, life habits, name, etc.

The analysis and consideration of these attitudes of professionals supporting migrants in educational, therapeutic, academic, or legal settings are essential in transcultural work. These attitudes are objectified only by third-party references (supervisors, institutions, teams, theoretical references, etc.) (14).

When developed, the question of the cultural countertransference most often allows cultural misunderstandings to be resolved. Thus, team discussions make it possible to discriminate the portion of subjectivity, and to take the woman’s own cultural representation into better account. Of course, the impact on interactions of postpartum depression, which is also more frequent in the migrant population, must not be underestimated. The point is to not induce pathological doubt in situations that do not fall in this category, and this requires reciprocal learning about cultural representations. To detect these attitudes, we propose that therapists engage in/commit to a process of decentering in relation to their own cultural affiliations. Clinicians must participate in the experience of cultural contacts, all the while creating methods to promote reflexivity concerning the intercultural dynamics that unfold during these contacts (14). The clinician’s cultural subjectivity can be revealed by debriefing at the end of the consultation between the different therapists involved in it.

In situations regularly encountered with migrants (especially those who have traversed conflicts or wars and/or been subjected to trauma during their voyage, often intentional and repetitive), a traumatic dimension of countertransference can provide information about the impact of this trauma. Attention must also be paid to the risk of vicarious trauma (80) that can engender stupefaction, a blank mind. You do not have to tell yourself that this story is impossible because it is beyond comprehension; on the contrary, it may well signify real traumatic events. Moreover, psychotrauma can cause memory disorders and confusion in the patient’s narrative: the description of dates and places can be complicated or inconsistent (81). Thus, knowledge of migrant journeys and elements of international politics/policy can be useful to therapists to avoid this pitfall of incredulity due to the unthinkable. Trauma implies precisely a form of insanity, in that it is defined by an experience of brutal and sudden confrontation with the reality of death (our own or that of others), stripped of all mediation of the signifying system that in ordinary life preserves the subject from the unexpected apprehension of nothingness (82).

While the patient’s own rhythm must be respected in managing psychotrauma, professionals must not be inhibited in their approach to some elements if its clinical consequence or functional discomfort is important. Sometimes, avoidance-type counter-transference reactions by care providers who are not psychologists or psychiatrists also protect against not knowing how to use a traumatic narrative/story for therapeutic purposes/what to do with it on a therapeutic level. Subjects such as excision or children conceived by rape, for example, can cause blockages because of a feeling of lack of know-how due to the cultural distance (71).

Finally, some studies show that the analysis of therapists’ countertransference allows a glimpse of what the babies may perceive through the mirror of their mothers (83), especially in traumatic dissociation.

### 4.2. Have detailed knowledge of the clinical aspects/presentation of complex trauma and of the cultural coding of symptoms of depression and anxiety

Numerous mothers and future mother present complex migration journeys, spattered with repeated traumatic events. The disorders observed often fit the clinical picture of a complex posttraumatic stress disorder (complex PTSD). Initially described by Herman (84), it is currently included in ICD 11 (11th revision of the World Health Organization’s International Classification of Diseases, that is, WHO ICD-11). Herman stressed the dissociative symptoms, the somatic manifestations, and the negative changes in self-perception (guilt, shame, modification of identity). Suspicion/distrust accompanied by impairment/changes of systems of meaning (despair and loss of fundamental beliefs) can impair relationships with others, including professionals.

The clinical picture of migrants is sometimes atypical and far from the classic reactivation syndrome. Profound and lasting personality changes have been described. Some symptoms associated with distrust may be interpreted as delusions of persecution, which would generate diagnostic errors (ref). Because of other conceptual models of *fright* in some societies, patients can make it into a narrative with spirits and ancestors, with coded cultural events, or represent themselves as singular beings who have overcome recurrent trauma.

Similarly, the experience of depression varies between cultures, negative affects can present as guilt feelings more prevalently in Western cultures and as feelings of shame or persecution more often in so-called “traditional” societies (85). Somatic symptoms can also be in the foreground in the expression of psychological distress (86), such as massive anxiety or depression.

In some situations of isolation, the absence of local peers can jeopardize the accuracy of this diagnostic evaluation. Active creativity is then called for. Because of the stakes of a diagnosis and from an ethical perspective: get information from an interpreter, agree to see someone close to the extended family or designated by the patient, even though they are not part of the family, make telephone calls to the country of origin… And especially do not turn too rapidly toward a stigmatizing diagnosis and a potentially inappropriate treatment.

### 4.3. Alliance and refusal of care

Most of the requests from hospital teams due to a refusal of care for apparent cultural issues involve a way of reclaiming the performative character of what is spoken, that is, ensuring that the patient’s discourse is heard and considered before any radical refusal is made.

Giving them a way to express their cultural representations can allow families to avoid western medical practices sometimes experienced as violent or immodest (delivery position, discarding the placenta, which can represents the baby’s real double, showing a photo of a dead fetus …). We can encourage respect for traditional means of protection, which are most often compatible with the medical care needed. Working on maternal competence assumes/presupposes taking into account their know-how about the rhythm of breastfeeding, solid food introduction with food from their native country, the importance of playing with the child, and how to raise a child or make him strong. Adopting a moralizing position risks breaking one’s bond with these patients, or even of undermining still more their sense of competence, already eroded by facing such different ways of doing things. The refusal of the cesarean described above is a good example of this. Among less extreme situations, let us mention the example of kaoline ingestion, frequent in West Africa: You can always inform women of the risk of anemia due to this consumption, consumption important to some women, because it is a common social practice in pregnancy. A refusal would most often reveal experience with a lack of consideration for what they have to say and with the other’s subjectivity. Negotiations are most often possible if they are explained after understanding the performative character of the other’s word. When a narrative can be developed, we often find an argument distant from the initial reason for refusal, more often associated with a singular aspect of the patient’s individual or family history. Identifying where the reluctance really comes from makes it possible to work better and overcome fears (Table 1).

**Table 1:** Summary: start working on cultural understanding early in the perinatal period- recommendations for health care workers (family doctors, psychologists, child psychiatrists, midwives, pediatricians, specialized child-care attendants, and social workers).

	Interview themes	Examples of content or questions
1	Start the interview by creating the basis for an appropriate relationship	Hospitality (Warmth and kindness) in the welcome Query the patient’s cultural identity with precision Arrange for an interpreter as soon as necessary Explain clearly the professional’s role and treatment objectives
2	Mitigate migration cleavage	Weave bridges between life there and life here Reestablish levels of continuity between here and there (religion, community, associative sectors)
3	Reconstruct the networks of belonging	What family remained there? Who is in France? How are they linked now (by telephone? how often?)? Did you announce the birth to them? Do you have friends, people from your cultural community nearby? Is the parent able sometimes to talk in her native language?
4	Help her to escape a feeling of powerlessness and facilitate her psychological reinvigoration	Help her capacities for resilience to emerge Show the heroic valence of the migration journey Help to coconstruct solutions: Even though she cannot do exactly as she would there, she can, for example, to perform a part of the ritual there. Or, do it here; it is equally functional even if it is not the day initially required.
5	Promote harmonious cultural métissage	Help her construct herself between the two cultural worlds she belongs to and take the creative richness of both (without it seeming inconciliable)
6	Construct a narrative of filiation	Historicize. include the child (or the future child) in the family history Ask about the baby’s name, for example (Co-)construct a genogram
7	Put the family in the position of the expert: Let them express themselves, listen to them fully	What is done in your cultural groups to welcome a child? What are the rituals around birth?
8	Elaborate a meaning	State the problem: How would you name it in your country of origin? Is there a related/relevant cultural therapeutic logic?
9	Stress the parents’ competence	Give yourself the means/resources (time, interpreter, team meetings) to avoid cultural misunderstandings. Welcome other ways of doing things Reinforce the parents’ feelings of competence

## 5. Conclusion

This article aims to help care providers to acquire/adopt transcultural skills, that is, an overall set of aptitudes and attitudes that allow them access to essential elements of their patient’s cultural understanding and that will help their patient to create bridges between their health care customs “back there” and here.

Transcultural clinical practices often ask us to be creative and to abandon the rigidity of some standard frameworks of care. It implies for the clinician, to adjust his overall approach, including his attitude, his health examination, his therapeutic framework, in order to access the patient’s cultural behaviors and mindsets. It also involves to overcome many relationships obstacles and to reduce the cultural distance which can both impact the therapeutic and diagnostic approach. Indeed, flexibility from health care workers promotes a thorough diagnostic evaluation and therefore a more effective therapeutic process, since the patient feeling more at ease and understood may be more inclined to share his distress, which can be a therapeutic process in itself by unlocking his normal thinking skills frozen by language barrier and cultural distance.

The transcultural approach is also a dynamic method that implies actively seeking levels of understanding, a general culture—learning from our patients and getting help from colleagues. It also supposes a form of sincere opening to the other’s culture and self-exposure, even while remaining professional. Every health care professional working in a cross-cultural world should not be scared of approaching cultural questions, by using an interpreter in situations where a language barrier exists, or by developing their openness and decentering skills. It will undoubtedly benefit the therapeutic alliance with the patient, particularly in the field of perinatal care in which cultural representations and native language play a crucial role.

## Ethics statement

Ethics approval was given by The “Comité d'Evaluation de l'Ethique des projets de Recherche Biomédicale (CEERB) Paris Nord” (Institutional Review Board (IRB) of Paris NorthHospitals, Paris 7 University, APHP). All participants were adult and provided written informed consent to participate in the study. Written informed consent for publication of any potentially identifiable data was obtained from all participants.

## Author contributions

RR, HC, JR, and MM made substantial contributions to the conception or design of the work, or the acquisition, analysis, or interpretation of data for the work. MB, HC, and AO drafted the work or revised it critically for important intellectual content. MR provided approval for publication of the content. All authors agreed to be accountable for all aspects of the work in ensuring that questions related to the accuracy or integrity of any part of the work are appropriately investigated and resolved.

## Conflict of interest

The authors declare that the research was conducted in the absence of any commercial or financial relationships that could be construed as a potential conflict of interest.

## Publisher’s note

All claims expressed in this article are solely those of the authors and do not necessarily represent those of their affiliated organizations, or those of the publisher, the editors and the reviewers. Any product that may be evaluated in this article, or claim that may be made by its manufacturer, is not guaranteed or endorsed by the publisher.

## Footnotes

^1^Insults or attitudes, "intentional or not" that "communicate hostile or contemptuous messages targeting people solely on the basis of their membership in a marginalized group."

^2^Tracing department and Restoring family links program provided by IRCR https://familylinks.icrc.org/

^3^A cultural transformation of identity that takes place in contact with the culture of the host country. The stress associated with the phenomenon of acculturation is recognized as a risk factor for the decompensation of several psychiatric disorders.

^4^Closure of the vulvar orifice during excision.

^5^Define maternal center.

## References

[1] Stork H. Enfances Indiennes. Etude de Psychologie Transculturelle et Comparée du Jeune Enfant. Paris: Paidos/Le Centurion (1988).

[2] Mesman J Basweti N Misati J. Sensitive infant caregiving among the rural Gusii in Kenya. Attach Hum Dev. (2018):1–9. doi: 10.1080/14616734.2018.1454053, PMID: 29564965

[3] Moro MR Réal I Baubet T. Consultation transculturelle en périnatalité: Une psychiatrie de liaison pour les bébés et leurs parents migrants In: Bailly D, editor. Pédopsychiatrie de Liaison: vers une Collaboration entre Pédiatres et Psychiatres. Rueil-Malmaison: Doin (2005). 35–41.

[4] Radjack R Guessoum S Ogrizek A Moro M. Prêter attention à la dimension culturelle en période périnatale pour mieux accompagner les parents et leurs bébés. Spirale. (2021) 99:73–81. doi: 10.3917/spi.099.0073

[5] Camara H Radjack R Klein A Di C Moro MR. Apprendre de la vie des mères. Approche Transculturelle Journal de la Psychanalyse de l’enfant. (2016) 6:151–72. doi: 10.3917/jpe.012.0151

[6] Kane H. Soins aux nouveau-nés: les recommandations internationales face aux enjeux sociaux de la naissance. Sante Publique. (2020) S1:17–27. doi: 10.3917/spub.200.0017 35724068

[7] Bowlby J. Attachement et Perte: la Perte, vol. 3. Paris: Puf (1978).

[8] Godelier M. Systèmes de parenté et formes de famille. Recherches de Science Religieuse. (2014) 102:357–72. doi: 10.3917/rsr.143.0357

[9] Fassin D Rechtman R. An anthropological hybrid: the pragmatic arrangement of universalism and culturalism in French mental health. Transcult Psychiatry. (2005) 42:347–66. doi: 10.1177/1363461505055620 16268233

[10] Sturm G. Culture, société, subjectivité: les innovations de l'ethnopsychanalyse file:///C:/Users/rahme/Downloads/healthcare-1952353.docxfrançaise In: Guerraoui Z, editor. Comprendre et Traiter les Situations Interculturelles: Approches Psychodynamiques et Psychanalytiques. Louvain-la-Neuve: De Boeck Supérieur (2011). 37–54.

[11] Moro MR. Guide de Psychothérapie Transculturelle: Soigner les Enfants et les Adolescents. Paris: Éditions in Press (2021).

[12] Mestre C. Psychothérapie transculturelle et institutionnelle In: . Bébés d'ici, Mères d'exil. Paris: Erès (2016). 155–88.

[13] Di C Bailly J Radjack R Moro MR. Éthique et soins: quand la clinique transculturelle ouvre sur des mondes. Ethics Med Public Health. (2020) 15:100603. doi: 10.1016/j.jemep.2020.100603hal-03493194

[14] Radjack R Ludot-Grégoire M Guessoum SB Moro MR. Évaluation clinique en situation transculturelle. EMC - Psychiatrie. (2022):1–10. doi: 10.1016/S0246-1072(22)44464-6

[15] Byers-Heinlein K Lew-Williams C. Bilingualism in the early years: what the science says. LEARNing Landsc. (2013) 7:95–112. doi: 10.36510/learnland.v7i1.632, PMID: 30288204 PMC6168212

[16] Truong M Paradies Y Priest N. Interventions to improve cultural competency in healthcare: a systematic review of reviews. BMC Health Serv Res. (2014) 14:99. doi: 10.1186/1472-6963-14-99 24589335 PMC3946184

[17] Kirmayer LJ Fung K Rousseau C Lo HT Menzies P Guzder J. Guidelines for training in cultural psychiatry. Can J Psychiatr. (2021) 66:195–246. doi: 10.1177/0706743720907505, PMID: 32345034 PMC7918872

[18] Okpaku SO. Therapeutic skills and therapeutic expectations in the treatment of migrant individuals and their families In: Bhugra D, editor. Oxford Textbook of Migrant Psychiatry. Oxford: Oxford University Press (2021). 467–74.

[19] Betancourt JR Green AR Carrillo JE Ananeh-Firempong O II. Defining cultural competence: a practical framework for addressing racial/ethnic disparities in health and health care. Pub Health Rep. (2003) 118:293–302. doi: 10.1016/S0033-3549(04)50253-4, PMID: 12815076 PMC1497553

[20] Goguikian Ratcliff B Diaz-Marchand N. Avoir un enfant loin des siens: petits gestes, grands enjeux In: . L'accompagnement des Familles: Entre Réparation et Créativité. Paris: L'Harmattan (2019)

[21] Mestre C. Bébés d'ici, Mères d'exil. Toulouse, France: Érès (2016).

[22] Van Gennep A. Les Rites de Passage: étude Systématique des rites de la porte et du seuil, de l’hospitalité, de l’adoption, de la grossesse et de l’accouchement, de la Naissance, de l’enfance, de la Puberté, de l’initiation, de l’ordination, du Couronnement des Fiançailles. In: Nourry, editors; (1909) (Vol. 5).

[23] Govindama Y. Temps et Rites de Passage: Naissance, Enfance, Culture et Religion. In: Karthala, editors; (2011).

[24] Carles G. Grossesse, accouchement et cultures: approche transculturelle de l’obstétrique. Journal de Gynécologie Obstétrique et Biologie de La Reproduction. (2014) 43:275–80. doi: 10.1016/j.jgyn.2013.12.002, PMID: 24440128

[25] Beninguisse G Nikièma B Fournier P Haddad S. L’accessibilité culturelle: une exigence de la qualité des services et soins obstétricaux en Afrique; (2005). Available at: https://tspace.library.utoronto.ca/bitstream/1807/5835/1/ep04044.pdf (Accessed December 31, 2004).

[26] Ben Mohammed K. Facteurs Psycho-socio-Culturels et Dépression Post-Natale en Tunisie: Exemple de la Prématurité [Thèse, Université Sorbonne Paris Nord et Université de Tunis]; (2010).

[27] Rodriguez J. L’expérience de la Césarienne chez les Femmes Migrantes: Une étude Qualitative Transculturelle. Paris: Mémoire de Master Recherche (2022).

[28] Lamèche R Sarraouinia G Radjack R. Vécu de la première expérience de grossesse et d’accouchement chez les femmes primo-arrivantes: une étude qualitative en Seine-Saint-Denis. Sages femmes. (2023) 22:23–26.

[29] Rapport final RéMI- Réduction de la mortalité infantile et périnatale en Seine-Saint-Denis. Equipe de recherche en Epidémiologie Obstétricale, Périnatale et Pédiatrique (EPOPé). ARS Ile-de-France; (2016). Available at: http://www.xn--epop-inserm-ebb.fr/wpcontent/uploads/2017/07/Rapport-REMIp_INSERM_Final-1.pdf. (Accessed September 04, 2022).

[30] Camara H Bossuroy M. Accompagnements psychologiques des femmes migrantes à l’hôpital dans la période périnatale In: Tunis BMGK, editor. Périnatalité: Parents, Bébés et Familles à Travers les Cultures. Tunis: Beit Al Hikma (2019)

[31] Ratcliff BG Sharapova A Suardi F Borel F. Factors associated with antenatal depression and obstetric complications in immigrant women in Geneva. Midwifery. (2015) 31:871–8. doi: 10.1016/j.midw.2015.04.010 26026198

[32] Stewart DE Gagnon A Saucier JF Wahoush O Dougherty G. Postpartum depression symptoms in newcomers. Can J Psychiatry. (2008) 53:121–4. doi: 10.1177/070674370805300208 PMID: 18357931

[33] Zelkowitz P Saucier JF Wang T Katofsky L Valenzuel M Westreich R. Stability and change in depressive symptoms from pregnancy to two months post-partum in childbearing immigrant women. Arch Womens Ment Health. (2008) 11:1–11. doi: 10.1007/s00737-008-0219-y, PMID: 18270652

[34] Mestre C Pinheiro L Guéry L. Affilier les enfants nés de viols politiques. L'Autre. (2022) 23:22–31. doi: 10.3917/lautr.067.0022

[35] Vandentorren S Le Méner E Oppenchaim N Arnaud A Jangal C Caum C. Characteristics and health of homeless families: the ENFAMS survey in the Paris region, France 2013. Eur J Pub Health. (2016) 26:71–6. doi: 10.1093/eurpub/ckv187, PMID: 26511600

[36] Weiss J. Female refugees face physical assault, exploitation and sexual harassment on their journey throught Europe In: . Sexual and Gender- Based Violence Prevention and Response in Refugee Situations in the Middle East and North Africa — Executive Summary. London: Amnesty International (2016)

[37] Mourouvaye Payet M Radjack R Moro MR. Les professionnels de maternité auprès des femmes migrantes, enceintes et à la rue. Soins Pédiatrie/Puériculture. (2019) 64:45–8. doi: 10.1016/j.soin.2019.04.012 31208583

[38] Panaccione E Moro MR. Construire de la sécurité dans l’errance. Maternité chez des femmes migrantes sans domicile fixe. Psychiatr Enfant. (2014) 57:533–61. doi: 10.3917/psye.572.0533

[39] Wing S. Microaggressions in Everyday Life. Race, Gender, and Sexual Orientation. New York: John Wiley & Sons (2010).

[40] Merry L Small R Blondel B Gagnon AJ. International migration and caesarean birth: a systematic review and meta-analysis. BMC Pregnancy Childbirth. (2013) 13:27. doi: 10.1186/1471-2393-13-27, PMID: 23360183 PMC3621213

[41] Urquia ML Glazier RH Mortensen L Nybo-Andersen AM Small R Davey MA. Severe maternal morbidity associated with maternal birthplace in three high-immigration settings. Eur J Pub Health. (2015) 25:620–5. doi: 10.1093/eurpub/cku230, PMID: 25587005 PMC4512956

[42] Gagnon AJ Zimbeck M Zeitlin J. Migration and perinatal health surveillance: an international Delphi survey. Eur J Obstet Gynecol Reproduct Biol. (2010) 149:37–43. doi: 10.1016/j.ejogrb.2009.12.002, PMID: 20074849

[43] Azria É. Précarité sociale et risque périnatal. Enfances Psy. (2015) 67:13–31. doi: 10.3917/ep.067.0013

[44] Sauvegrain P Stewart Z Gonthier C Saurel-Cubizolles M-J Saucedo M Deneux-Tharaux C. Accès aux soins prénatals et santé maternelle des femmes immigrées. Bull Epidemiol Hebd. (2017):389–95.

[45] Herschkorn BP. Parcours de soins périnatals et grande précarité: expérience du réseau SOLIPAM (Solidarité Paris Maman Île-de-France). Contraste. (2017) 46:189–206. doi: 10.3917/cont.046.0189

[46] Rietsch M-G. Rapport Final Issu du Projet de Recherche Commun. (2014).

[47] Radjack R Moro MR. Managing encounters with otherness - transcultural approaches in a French maternity unit In: Welsh G Moro MR, editors. Parenthood and Immigration in Psychoanalysis: Shaping the Therapeutic Setting. London (Great Britain): Routledge (2021)

[48] Gabai N Furtos J Maggi-Perpoint C Gansel Y Danet F Monloubou C. Migration et soins périnataux: une approche transculturelle de la rencontre soignants/soignés. Médecine Hygiène Devenir. (2013) 25:285–307. doi: 10.3917/dev.134.0285

[49] Bossuroy M Serbandini N Drain É Baubet T Ayosso J. Autour de l’accueil du bébé: intérêt d’une consultation transculturelle en maternité. Psychiatr Enfant. (2014) 57:563. doi: 10.3917/psye.572.0563

[50] Devereux G. (1972) Ethnopsychanalyse Complémentariste. Paris: Flammarion, Réédition (1985).

[51] Tervalon M Murray-Garcia J. Cultural humility versus cultural competence. A critical distinction in defining physician training outcomes in multicultural education. J Health Care Poor Underserved. (1998) 9:117–25. doi: 10.1353/hpu.2010.0233, PMID: 10073197

[52] Radjack R Touhami F Woestelandt L Minassian S Lachal J Mouchenik Y. Cultural competencies of professionals working with unaccompanied minors: Adressing empathy by a shared narrative. A qualitative study. Front Psychiatry. (2020) 11:528. doi: 10.3389/fpsyt.2020.00528, PMID: 32595535 PMC7301836

[53] Touhami F Minassian S Radjack R Moro MR. Le dessin, support de soins aux mineurs isolés étrangers. Soins Pédiatrie. (2017) 294:32–5. doi: 10.1016/j.spp.2016.11.007 28104269

[54] Ricoeur P. Soi-même Comme un Autre. Paris: Seuil (1990).

[55] Lachal J Escaich M Bouznah S Rousselle C De Lonlay P Canoui P. Transcultural mediation programme in a paediatric hospital in France: qualitative and quantitative study of participants’ experience and impact on hospital costs. BMJ Open. (2019) 9:e032498. doi: 10.1136/bmjopen-2019-032498, PMID: 31753892 PMC6887050

[56] Derivois D. Clinique de la mondialité. Vivre ensemble avec soi-même, vivre ensemble avec les autres. Louvain-la-Neuve: De Boeck (2017).

[57] Guedeney N Guedeney A. L’attachement: Concepts et Applications. Paris: Masson (2006). 160 p.

[58] La NT. Folie des Autres. Traité D’ethnopsychiatrie Clinique. Paris: Dunod (1986).

[59] Chandler M Lalonde C. Cultural continuity as a hedge against suicide in Canada’s first nations. Transcult Psychiatry. (1998) 35:191–219. doi: 10.1177/136346159803500202

[60] Aulagnier P. Se construire un passé. Journal de Psychanalyse de l’enfant. (1989) 7:119–220.

[61] Prevost C Drain E Carbillon L Taieb O Baubet T. Etre mère ici et là-bas, une parentalité complexe. L’Autre. (2021) 22:61–70. doi: 10.3917/lautr.064.0061

[62] Camara M Seck S Ba E Faye P Thiam M. Le confiage: mécanismes et enjeux relationnels. L'Autre. (2014) 15:167–77. doi: 10.3917/lautr.044.0167

[63] Knipiler T. Vécu et Représentations Parentales de la Place de la Famille Élargie Après une Naissance très Prématurée. Paris: Thèse Psychiatrie (2017).

[64] Woestelandt L. Initier un Suivi Psychiatrique chez les Jeunes Isolés Étrangers: Revue de la Littérature et Apport d’une Recherche Qualitative à la Compréhension de la Place des Soins. Paris: Thèse Psychiatrie (2016).

[65] Selten J-P Van der Ven E Termorshuizen F. Migration and psychosis: a meta-analysis of incidence studies. Psychol Med. (2020) 50:303–13. doi: 10.1017/S0033291719000035, PMID: 30722795 PMC7083571

[66] Tallarico S. Quand les Esprits Troublent les Esprits; Patients et Cliniciens à la Rencontre du Monde Invisible. Paris: Thèse de Psychologie (2019).

[67] Golse B Moro MR. Le concept de filiation narrative: un quatrième axe de la filiation. Psychiatr Enfant. (2017) 60:3–24. doi: 10.3917/psye.601.0003

[68] Bossuroy M Camara H. La clinique transculturelle en périnatalité aujourd’hui In: Mouchenik Y Moro MR, editors. Pratiques Transculturelles: Les Nouveaux Champs de la Clinique. Paris: Editions in Press (2021)

[69] Cautru F. Accoucher prématurément en exil: histoires d’accompagnement à la parentalité. Le Carnet PSY. (2022) 5:24–6.

[70] Camara H Moro MR. L’éveil du tout petit dans d’autres cultures. Cahiers de Puériculture. (2015) 52:24–5. doi: 10.1016/j.cahpu.2015.05.002

[71] Radjack R Molino L Ogrizek A Ngameni EG Moro MR. How do we address and treat the trauma of a 16-year-old girl, unaccompanied minor, and her rape-born son? A case report. Healthcare. (2022) 10:2036. doi: 10.3390/healthcare10102036, PMID: 36292484 PMC9602657

[72] Rizzi AT Moro MR. Le génogramme transculturel. In Mouchenik Y. Moro MR. (dirs.). Pratiques Transculturelles: Les Nouveaux Champs de la Clinique. Paris: Editions In Press (2021).

[73] De DG. L’angoisse à la Méthode Dans les Sciences du Comportement (1967). Paris, France: Flammarion (1980).

[74] Heidenreiche F. Sur la corde raide - les étiologies "traditionnelles" et leur utilisation dans le dispositif ethnopsychiatrique. L'autre. (2010) 11:199–206. doi: 10.3917/lautr.032.0199

[75] Le ZA. sens de l'insensé: de l'interprétation « magico-religieuse » des troubles psychiques. Psychiatrie Française. (1983) 4:29–47.

[76] Perrin AS Drain E Sarot A Moro MR. Comment soutenir l’arrivée au monde d’un enfant de mère migrante dans une maternité française: entre urgence somatique et urgence psychiatrique, le temps de la reconstruction. Neuropsychiatr Enfance Adolesc. (2016) 64:31–5. doi: 10.1016/j.neurenf.2015.11.004

[77] Aziken M Omo-Aghoja L Okonofua F. Perceptions and attitudes of pregnant women towards caesarean section in urban Nigeria. Acta Obstet Gynecol Scand. (2007) 86:42–7. doi: 10.1080/00016340600994950 17230288

[78] Rouchon JF Reyre A Taïeb O Moro MR. L'utilisation de la notion de contre-transfert culturel en clinique. L'Autre. (2009) 10:80–9. doi: 10.3917/lautr.028.0080

[79] Azria E Sauvegrain P Anselem O Bonnet MP Deneux-Tharaux C Rousseau A. Implicit biases and differential perinatal care for migrant women: methodological framework and study protocol of the BiP study part 3. J Gynecol Obstet Hum Reprod. (2022) 51:102340. doi: 10.1016/j.jogoh.2022.102340, PMID: 35181544

[80] Lachal C. Comment se Transmettent les traumas? Traumas, Contre-Transferts, Empathie et Scénarios Émergents. Grenoble: La Pensée Sauvage (2015).

[81] Baubet T Moro MR. Psychopathologie Transculturelle. Paris: Masson (2013).

[82] Lebigot F. Traiter les Traumatismes Psychiques, Clinique et Prise en Charge. Paris: Dunod (2011).

[83] Feldman M El Husseini M Dozio E Drain E Radjack R Moro MR. The transmission of trauma from mother to infant: radioactive residues and counter-transference in the case of a Haitian mother and her two-year old son. Child Care Pract. (2019) 25:215–226. doi: 10.1080/13575279.2017.1342600

[84] Herman J. Complex PTSD: a syndrome in survivors of prolonged and repeated trauma. J Trauma Stress. (1992) 5:377–91. doi: 10.1002/jts.2490050305

[85] Kirmayer LJ Guzder J Rousseau C eds. Cultural Consultation: Encountering the Other in Mental Health Care. Berlin, Germany: Springer Science & Business Media (2013).

[86] Ludot-Grégoire M Harf A Ibrahim N Merlo M Hassler C Rietsch J. Bodily expression of psychological distress in adolescents: a qualitative study. Child Adolesc Psychiatry Ment Health. (2022) 16:40. doi: 10.1186/s13034-022-00476-9, PMID: 35659270 PMC9166518

